# Graph representation learning for structural proteomics

**DOI:** 10.1042/ETLS20210225

**Published:** 2021-10-19

**Authors:** Romanos Fasoulis, Georgios Paliouras, Lydia E. Kavraki

**Affiliations:** 1 Department of Computer Science, Rice University, Houston, TX, U.S.A.; 2 Institute of Informatics and Telecommunications, NCSR Demokritos, Athens, Greece

**Keywords:** protein structure, proteomics, deep learning, machine learning, graphs, graph learning

## Abstract

The field of structural proteomics, which is focused on studying the structure–function relationship of proteins and protein complexes, is experiencing rapid growth. Since the early 2000s, structural databases such as the Protein Data Bank are storing increasing amounts of protein structural data, in addition to modeled structures becoming increasingly available. This, combined with the recent advances in graph-based machine-learning models, enables the use of protein structural data in predictive models, with the goal of creating tools that will advance our understanding of protein function. Similar to using graph learning tools to molecular graphs, which currently undergo rapid development, there is also an increasing trend in using graph learning approaches on protein structures. In this short review paper, we survey studies that use graph learning techniques on proteins, and examine their successes and shortcomings, while also discussing future directions.

## Introduction

Proteins are the building blocks of all cells in our bodies. Although the DNA molecule holds all the information that is necessary for life, it is proteins that carry out what is coded in the genetic material [1]. As protein function is largely determined by its three-dimensional (3D) conformation, knowing the tertiary structure of a protein is a basic prerequisite for understanding its function [2]. While many specialized protein structural databases exist [3,4], the Protein Data Bank (PDB) is the de facto internationally recognized repository that holds determined experimentally 3D protein structures [5]. In the last two decades, we have seen a substantial increase in protein structures deposited in PDB [6], as well as an increase in its usage by scientists in the field [7]. Additionally, as a result of the success of Alphafold [8,9] models in predicting protein structures from their amino acid sequence, a large database was recently created, holding modeled structures of almost the entire human proteome [10].

Parallel to the increase in structural data in the field of biology, novel machine learning (ML) and deep learning (DL) approaches are being developed that can harness huge amounts of data to achieve high predictive performance [11,12]. In the last few years, increasing efforts have been made to expand DL techniques to the geometrical domain, in order to learn from complex structural data, particularly in tasks where the structural component is strong. As a result, the umbrella term *geometric deep learning* was created, encompassing these techniques [13], a subset of which comprises graph learning models that are used for modeling network relations, data-induced similarities, as well as 3D shapes [13]. Graph-based learning approaches have received praise and have achieved great results on benchmark network datasets, thus, encouraging researchers to employ those methods in different domains and applications. Graph based models have been used in recommender systems, social networks, materials research and others [14]. Graph learning models have also been employed in biological fields, with one of the most recent, bio-related successes involving molecular graph learning, a subfield in which graph learning models are used for predicting biochemical properties of molecules. Advances in this field resulted in both developing molecule-specific graph models that are more specialized in extracting/using molecular structure information [15] and advancing the graph learning field as a whole as well [16].

Given the increase in protein structural data and the success of graph learning methods, it is natural for studies that are employing graph learning models in the structural proteomics field to emerge. The goals of this short review are to:

- Provide related work on protein graph-based representations.
- Introduce the graph representation learning (GRL) field, and explore its potential use to structural proteomics.
- Report studies in six different proteomics task categories where graph learning models have been successfully used.

## Proteins as graphs

The graph representation of a protein structure collapses its 3D conformation into a graph, where now, the geometric information is incorporated within the graph connectivity, and not explicitly encoded in a coordinate system. Nodes in the graph can be defined at an amino acid level, where each node corresponds to a different amino acid in the protein sequence. Biochemical features for each node include can polarity, charge, hydrophobicity and others [17]. Protein graphs can also be defined at an atom level, where each node corresponds to an individual atom, and node features include the atom type and charge. The node-level representation of choice depends on the application. Atom-level protein graphs can, in principle, be more expressive, but the computational cost of working with a larger graph must be taken into account.

**Fig. 1.**
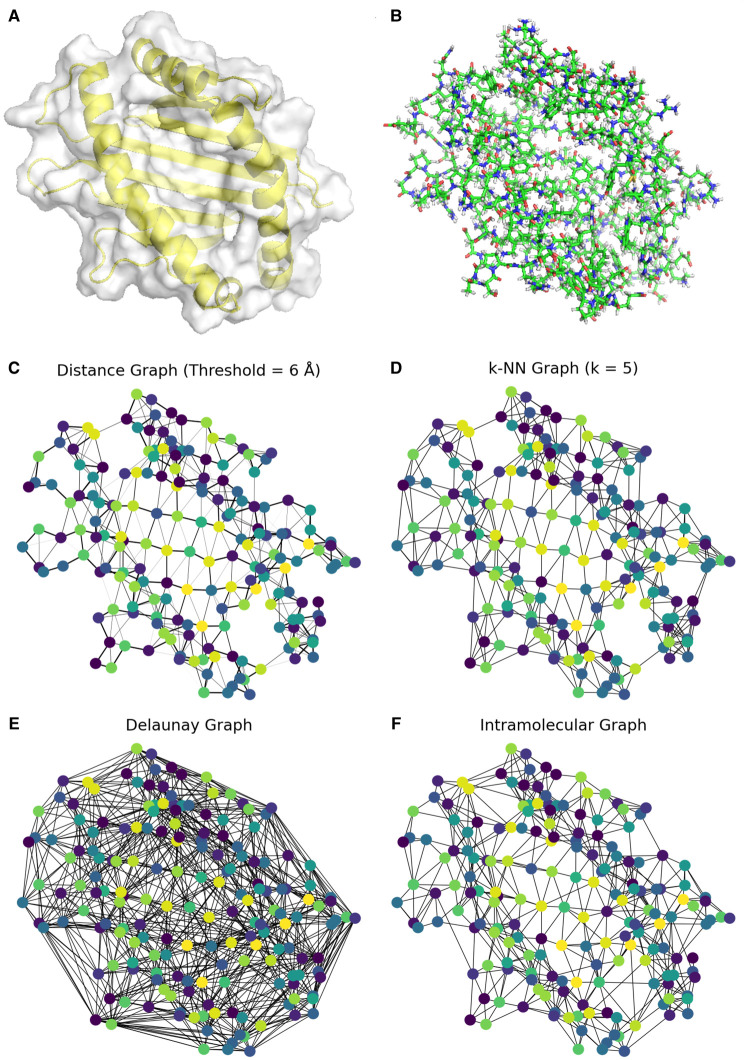
Graphical representation of the binding interface of HLA-B*52:01 (PDB: 3W39, peptide removed). Graphs are shown at an amino-acid level, each color corresponding to a different amino acid, and geometrical information is not expressed in 3D coordinates, but as relations/edges in the graph. (**A**) The 3D cartoon model, highlighted in yellow color, while the surface is shown in white color. (**B**) The 3D all-atom model, represented as sticks. (**C**) The distance graph, where each edge between two residues denotes that their actual distance is smaller than a cutoff δ (here equal to 6 Å). For calculating pairwise distances, α-carbon atoms are used as centroids. (**D**) The k-NN graph, where each residue is forced to connect to its k=5 closest residue neighbors. (**E**) The Delaunay graph, created using Delaunay triangulation. (**F**) The intramolecular graph; each edge denotes a chemical bond (covalent/non-covalent).

However, transforming a protein tertiary structure to a graph is far from trivial, as different topological and geometric information can be taken into account in the process. As such, different geometrical/biological methods arise for creating the graph. A simple way is to calculate all residue pairs distances, and introduce an edge between two residues if the distance is smaller than an empirically proposed cutoff δ. To avoid over-connectivity or exceeding sparsity, a k-nearest neighbors approach can also be used, where each node is connected to its k less distant neighbors. However, it is likely that this simple discretization does not capture accurately the geometrical structure of the underlying topological manifold [13]. Many studies have experimented with performing Delaunay triangulation (the dual graph of the Voronoi diagram), which is able to extract hierarchical molecular information about protein structure [18,19]. Such information though can result in a denser graph [20] (Figure 1C), possibly dilating useful information for the task at hand [21]. Computational tools have been developed that identify an edge in the graph as an existing intramolecular interaction [20], (i.e. hydrogen bonds, salt bridges, pi-cation bonds) providing additional useful biochemical information. A visual review of the methods discussed above can be seen in Figure 1.

Graph representations have been extensively used in protein analysis. The Gaussian network model [22,23] and the anisotropic network model [24,25] model the protein as an elastic network with 3D derived topological constraints, and have been pivotal in studying protein dynamics and flexibility. Graph-theory approaches have also been used for a variety of tasks, from protein structure identification [26], to side-chain prediction [27], among others (see [28,29] for graph theory methods in proteomics tasks). However, many of the aforementioned methods rely on theoretical techniques and models that are not specific to proteomics, while others incorporate empirical knowledge and constraints that may not be applicable to the task at hand. With the rise of graph learning and the increasing amount of protein structural data, an alternative approach becomes feasible; namely, one that relies on algorithms that learn the relevant structural information from graph data. In the following sections, we will describe the rising field of GRL and the way that knowledge can be learned from graph data in an end-to-end fashion.

## Graph representation learning

### Learning from graph structure

DL approaches have shown state-of-the-art results, partly due to their increased representational capacity [12], but also due to the inductive bias of the DL architectures themselves [30]. This is particularly true for convolutional neural networks (CNNs), which are very effective in processing image data, due to their locality and translational invariance [31]. Given their success, CNN architectures have been applied to more general, non grid-based data [13].

In this context, the emerging field of GRL seeks to represent graph data in such a way that it can be given as input to a standard neural network-like architecture for further downstream tasks. More formally, the idea is to learn a mapping function that compresses node, sub-graph, or entire graph information to a vector of fixed dimensions. This vector, containing not only node/graph attribute information, but also structural information stemming from the graph connectivity itself, can be used as a feature input for downstream tasks [32].

While embedding nodes and graphs goes far back [33,34], during the last few years, GRL has dominated the field, demonstrating state-of-the-art results in numerous graph-related tasks, such as node classification, link prediction, and graph classification [35]. One of the reasons for this is that more recent GRL approaches integrate the feature extraction and training processes in an end-to-end manner. In this way, they learn the appropriate graph embeddings for the task at hand, in contrast with methods that extract graph information in a task-agnostic way [36]. One of the most popular GRL methods, capable of achieving end-to-end learning, is the graph neural network (GNN).

### Graph neural networks

GNNs appeared more than 10 years ago [37,38], and different variations were slowly surfacing [39,40]. However, GNNs became mainstream after the introduction of simplified operations, more specifically, after the seminal graph convolutional network (GCN) paper in 2017 [41]. Since then, the field has exploded, with different GNN models and extensions having various applications to many different domains [14].

**Fig. 2.**
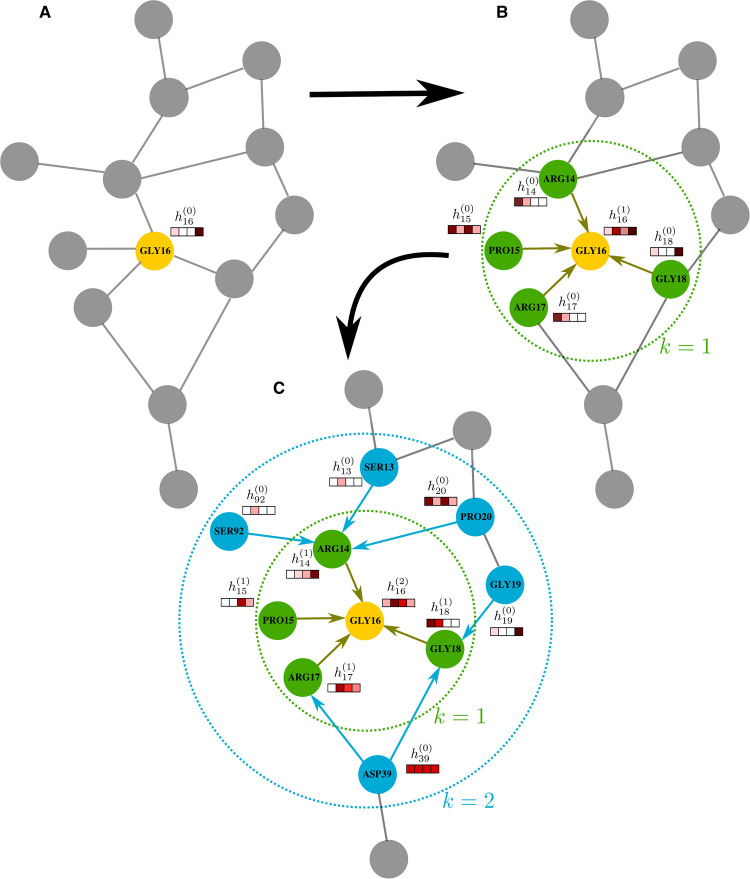
Convolution operations performed on a protein subgraph. (**A**) The residue of interest here is GLY16, and $h_{16}^{\left(0\right)}=x_{16}$ represents its biochemical features. (**B**) The first GNN layer. Biochemical features from residues belonging to GLY16’s immediate neighbors are aggregated and transformed in order to calculate the embedding $h_{16}^{\left(1\right)}$, injecting topological information in the process. (**C**) Multiple convolutional operations can be used in series to take into account information from distant neighbors. This however can potentially cause *oversmoothing* on the GLY16 embedding [42].

Consider a protein graph G, comprised of residue nodes V and edges E, denoted as G(V,E). The set of edges E will be different for different transformations of a protein structure to a graph (Figure 1). Each residue i has a set of biochemical features $x_{i}$. The set of neighbors of a residue i is denoted as $N_{i}$. GNNs use the following operator to calculate an embedding representation $h_{i}$ of a node i at layer k of the network (see Figure 2 for a visual representation):

1 $$h_{i}^{\left(k\right)}=\text{UPDATE}(h_{i}^{\left(k-1\right)},\;\text{AGGREGATE}(\{h_{u}^{\left(k-1\right)}\;|\;u\in N_{i}\})),\;h_{i}^{\left(0\right)}=x_{i}$$

What is achieved with the above operator for each node/residue is the transformation from a graph domain representation to a vector representation, while still retaining topological information. After the GNN operator is applied, a residue i will not be characterized only by its biochemical features $x_{i}$, but its embedding $h_{i}$ will also contain biochemical information from its topological neighborhood. Many layers can be put together in series, so that information from distant neighbors can be obtained (Figure 2C). However, careful experimental selection of hyperparameters and number of GNN layers is crucial. Too much information from distant neighbors being aggregated into a single embedding can result in over-smoothing/over-squashing the important biological information that distinguishes each residue [21,42].

It is worth noting that equation (1) serves as a blueprint to design and create GNN models. There are many GNN variations [14,35], instantiating the UPDATE and AGGREGATE functions. In other words, they differ in how neighborhood information of a node i is aggregated, and how it is combined with its representation $h_{i}$ [43]. For example, for a given node i, GCN’s [41] AGGREGATE function averages and normalizes element-wise all neighbor embeddings (including the embedding of node i itself using self-connections). To UPDATE the new representation $h_{i}^{\left(k\right)}$ at layer k, the result from the AGGREGATE function passes through a simple one-layer feed forward neural network [12]. Hence, GCN calculates an embedding representation as follows:

2 $$h_{i}^{\left(k\right)}=\sigma \left(W^{k}\sum_{u\in N_{i}\cup i}^{}\frac{1}{\sqrt{|N_{u}||N_{i}|}}h_{u}^{\left(k-1\right)}\right),\;h_{i}^{\left(0\right)}=x_{i}$$

Another popular variant of GNNs is the graph attention network (GAT) [44], which is highly used in structural proteomics tasks (Table 1), due to its ability to select the important residue/atom neighbors for a given node. While GATs UPDATE function remains largely the same as GCNs, its AGGREGATE function differs from GCNs in that the averaging is weighted, with the weights $a_{u,i}$ being learned from data:

3 $$h_{i}^{\left(k\right)}=\sigma \left(W^{k}\sum_{u\in N_{i}\cup i}^{}a_{u,i}h_{u}^{\left(k-1\right)}\right),\;h_{i}^{\left(0\right)}=x_{i}$$

There are numerous review papers on explaining and analyzing different GNN models, and presenting them in detail is out of the scope of this paper ([14,35] provide a review).

**Table 1.** List of recent graph-learning-based methods for structural proteomics tasks

Category	Study	Graph type	Edge type	GNN layer type	Year	Reference
Protein–ligand prediction	GraphBAR	Atom graph (protein–ligand)	Distance edges (protein–ligand)	GCN-based	2021	[45]
	Lim et al.	Atom graph (protein–ligand) + atom graph (protein) + atom graph (ligand)	Distance edges (protein–ligand) + chemical bonds (protein) + chemical bonds (ligand)	GAT-based [44]	2019	[46]
	Torng et al.	Residue graph (protein) + atom graph (ligand)	Distance edges (protein) + chemical bonds (ligand)	GAE-based [47] + Duvenaud et al. [48]	2019	[49]
	DGraphDTA	Residue graph (protein) + atom graph (ligand)	Distance edges (protein) + chemical bonds (ligand)	GCN-based	2020	[50]
	GEFA	Residue graph (protein) + atom graph (ligand)	Distance edges (protein) + chemical bonds (ligand)	GCN-based + skip connections [51]	2020	[52]
Binding site identification	Fout et al.	Residue graph (proteins)	k-NN edges (proteins)	GCN-based + edge features	2017	[53]
	PECAN	Residue graph (proteins)	Distance edges (proteins)	GCN-based	2020	[54]
	PepNN	Residue graph (proteins)	k-NN edges (proteins)	Graph transformer [55]	2021	[56]
Docking scoring	EGCN	Residue graph (protein–ligand)	Distance edges (protein–ligand)	GCN-based + edge features	2020	[57]
	GNN-DOVE	Atom graph (protein–ligand) + atom graph (protein) + atom graph (ligand)	Distance edges (protein–ligand) + chemical bonds (protein) + chemical bonds (ligand)	GAT-based	2021	[58]
	InterPepRank	Residue graph (protein–ligand)	Distance edges (protein–ligand) + chemical bonds (protein–ligand)	Simonovsky et al. [59]	2020	[60]
Protein model quality assessment	GraphQA	Residue graph (protein)	Distance edges (protein) + covalent bonds (protein)	Graph Nets [31]	2021	[61]
	ProteinGCN	Atom graph (protein)	k-NN edges (protein)	Xie et al. [62]	2020	[63]
	VoroCNN	Atom graph (protein)	Voronota [64] edges (protein) + chemical bonds (protein)	GCN-based	2021	[65]
	S-GCN	Residue graph (protein)	Voronota edges (protein)	Custom	2020	[66]
Protein function prediction	DeepFRI	Residue graph (protein)	Distance edges (protein)	GAT-based	2021	[67]
	PersGNN	Residue graph (protein)	Distance edges (protein)	GCN-based	2020	[68]
	Gelman et al.	Residue graph (protein)	Distance edges (protein)	GCN-based	2021	[69]
Protein design	ProteinSolver	Residue graph (protein)	Distance edges (protein)	Wang et al. [70]	2020	[71]
	MimNet	Residue graph (protein)	Distance edges (protein)	GCN-based + U-net [72]	2021	[73]
	Ingraham et al.	Residue graph (protein)	k-NN edges (protein)	Graph transformer	2019	[74]

Protein topology has a central role in many biological processes. Therefore, GNNs, and the GRL field as a whole, being able to harness topological information, can potentially be a great tool in advancing the structural proteomics field. In the next few sections, we will examine GRL approaches that have been used for six proteomics-related tasks (Table 1).

## Protein–ligand interaction

Protein–ligand interaction has been a well-studied problem. The underlying physicochemical mechanisms, i.e., binding kinetics, basic thermodynamic concepts, and the binding driving forces/factors have been extensively studied [75]. However, the way that those mechanisms intersect and contribute to the binding is very complex. Instead of trying to explain the interactions in depth, the increase in structural data allows graph-based methods to learn from data and provide accurate protein–ligand interaction predictions.

**Fig. 3.**
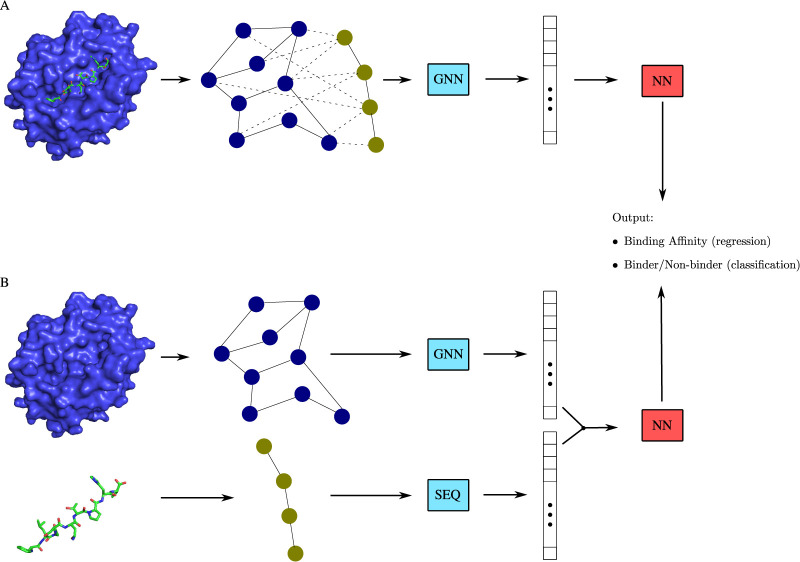
Protein–ligand interaction studies using GNNs. (**A**) Given a sufficient amount of protein–ligand structures, the graph of the whole complex can be given as an input to a GNN architecture. (**B**) Even when peptide–ligand complex data are not available, the protein complex and the ligand sequence can be processed as separate entities, retaining structural information during learning.

Studies have shown good drug-target affinity predictions when training a GNN on a sufficient amount of protein–ligand complex data (Figure 3A), and one such method is GraphBAR, a graph DL-based binding affinity prediction model [45]. GraphBAR represents the whole protein–ligand complex as a graph of connected atoms, that combines multiple adjacency matrices based on different distance measures, together with a feature matrix providing the molecular properties of the atoms. Graph convolutional operators are used to encode topological information of the complex, leading to superior performance over a CNN model for protein–ligand binding affinity prediction. In [46], a graph model is built that takes the atom graph representation of the protein–ligand binding pose to classify compounds as active or inactive. Two atom graphs are constructed, one including only covalent bonds, and one including both covalent/non-covalent bonds, and GAT [44] architectures are employed on both graphs to aggregate/differentiate between different molecular bonds. The paper reported improved performance compared with docking methods and other DL approaches.

GRL methods have been used even when protein–ligand complex data are not available. The corresponding studies treat the ligand and the protein as separate entities, with ligands being represented as small atom graphs, and proteins as large residue or atom-based graphs (Figure 3B). The authors of [49] use two separate GCNs to extract features from the protein binding pocket graph and the ligand atom graph. Their approach outperforms 3D-CNNs on virtual screening, while also producing interpretable results that indicate the contributions of each ligand atom and pocket residue to the classification decision. DGraphDTA [50] and GEFA [52] are methods that improve upon the earlier GraphDTA [76], which combines an atom-based graph representation for the ligand and a sequence representation for the protein. DGraphDTA first predicts the protein contact map based on the protein’s amino acid sequence, and then it constructs a protein graph given the contact map. GCN architectures are used for both the protein residue graph and the ligand atom graph. GEFA introduces an early protein–ligand fusion approach, where, an attention-based mechanism connects the drug to the protein in a learnable fashion, and the unified protein–ligand representation passes through a GCN layer to predict drug-target affinities. Both studies achieve superior drug-target affinity predictions to GraphDTA and other purely sequence-based DL architectures like DeepDTA [77].

As the number of datasets and studies that GNN-based methods are tested is still low, it is unknown how well the methods discussed above generalize. Moreover, in the case where the protein–ligand complex is not given (Figure 3B), there is no sufficient evidence that the contributions of the ligand atoms presented in [49] or the protein–ligand connections learned in [52] correlate with actual structural protein–ligand data. However, it is evident that — whether there is enough protein–ligand complex data or not-using a graph representation and applying GNN-based architectures can improve binding affinity predictions compared with CNN-like approaches and sequence-based models [46,50].

## Binding site identification

In a protein interaction, it is not only valuable to know the binding affinity, but also, the components that determine the interaction, i.e. the binding sites. In one of the first studies to use GRL approaches to structural proteomics, a protein–protein interaction system based on multiple stacked layers of graph convolutions was developed [53]. Given a pair of amino-acid residues from two different proteins, the system predicts whether the amino-acids will interact. The proposed method was shown to outperform a simpler method not based on graph convolutions, indicating that the use of information from a residue’s neighbors improves the accuracy of interface prediction. PECAN employs a similar type of framework, employing two GCNs and an attention module to assign each node of the primary structure a probability of belonging to the binding interface [54]. In a more recent work, the authors at [56] developed two different peptide-binding site identification models, one based on structure (PepNN-Struct) and one on sequence (PepNN-Seq). Similarly to [53], they showed that incorporating structure and encoding it as a graph improves performance on different test sets, showcasing the potential of GNN-based approaches to binding site identification.

## Scoring for docking

**Fig. 4.**
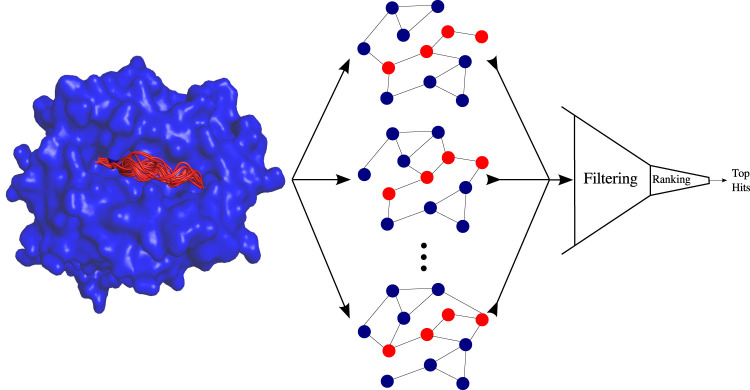
Proof-of-concept pipeline for protein-peptide docking scoring using GNNs. The filtering and ranking system, based on graph message passing GNN modules, takes graph conformations as input, and lists the top scoring ones.

The 3D conformation of a receptor–ligand complex can affect specific biologically related functions, such as driving the cellular immune response [78]. To this end, various computational approaches have been developed that predict the 3D conformation of a ligand to a receptor [79–81]. Docking consists of sampling candidate conformations of the ligand and scoring them. GRL approaches have been proposed for ranking candidate protein–peptide and protein–protein conformations (see Figure 4 for an illustration of a protein–peptide docking scoring system). InterPepRank [60] generates a large dataset of protein–peptide complex decoys using FFT-docking [82] and then trains a GCN to predict the ligand root-mean-square deviation of decoys through edge-conditioned graph convolutions. In the context of protein–protein scoring, EGCN [57] uses a deep graph learning model to rank protein–protein docking models. EGCN significantly improves the ranking for a CAPRI test set involving homology docking. Finally, similarly to [46], GNN-DOVE [58] builds two different graphs, one using only the covalent bonds of the protein–protein pair, and one that considers non-covalent, distance based connections. GNN-DOVE uses both to classify whether a decoy is correct or not.

Overall, GNNs seem to show promise in regards to scoring conformations. However, it is questionable how well receptor–ligand graphs with microscopic structural differences can be differentiated with a GNN for scoring. Additionally, no extensive tests have been performed with ML-based scoring functions that use more fine-grained structural features than the whole receptor–ligand graph [83].

## Protein model quality assessment

Predicting the 3D structure of a protein given its sequence is one of the most challenging problems in the field of computational biology [84]. Despite the undoubtedly great advances that Alphafold [8,9] has achieved in the recent critical assessment of protein structure prediction (CASP) challenges, the problem is still far from being solved, as the accuracy of predictions can still vary significantly [66]. Therefore, estimating the reliability of a modeled structure is very important. This task is known as protein model quality assessment (QA), and it is one of the sub-challenges of CASP [85]. What QA boils down to is scoring, both locally (per residue) and globally (whole complex), how reliable a modeled structure is, a very helpful metric when there is no experimental structure available.

In line with graph-based methods exploding in popularity, GNN-based approaches have also been tested in QA, with GraphQA [61], based on Graph Nets [31], being one of them. GraphQA, using a residue graph representation of the protein, achieves increased performance on both local and global quality assessment tasks in comparison with other QA methods [61]. ProteinGCN [63] is similar to GraphQA, but differs in the representation of the protein graph -which is done at an atom-level- and the convolutional operators [62]. While ProteinGCN has not been compared direclty to GraphQA, increased performance to state-of-the-art QA methods is reported [63]. Instead of computing the protein graph through a residue distance like in GraphQA or through a k-nearest-neighbor approach like in ProteinGCN, VoroCNN [65] builds a graph through the Voronoi 3D tessellation of a protein 3D model. Using the resulting graph, a GNN predicts local qualities of 3D protein folds. Finally, S-GCN [66] is a different convolution operator based on spherical harmonics, which is more suited in exploiting geometrical and topological information of the protein graph, achieving superior performance to state-of-the-art QA methods.

The abundance of different graph methods, as well as the increased performance gains in comparison to other methods, indicate that GNN-based architectures are well suited to the QA task. As the methods presented above employ different graph convolution architectures and graph representations, future work includes benchmarking those methods on different datasets to further investigate which graph representations and architectures work best.

## Protein function prediction

Protein function prediction is a challenging task that is approached by various sequence-based and structural-based methods [86]. However, the fact that the function of a protein is intrinsically related to its 3D conformation (more than to its primary sequence) motivates the use of structure in predicting protein function. Many studies have used a graph representation of the protein, indicative of its geometry and topology, to predict protein function. DeepFRI [67] uses GNNs to predict protein function, by leveraging both sequence features extracted from a protein language model and protein structures. DeepFRI achieved better performance than its CNN predecessor DeepGO [87] which mainly uses protein sequence data. PersGNN [68] is another hybrid model that uses GNN operators, combined with persistent homology [88] to outperform simple neural-networks and vanilla GNNs on protein function prediction. The extensive benchmarking of GNN models in ENZYMES [89], PROTEINS [90] and D&D (Dobson and Doig) [91] datasets demonstrates the good performance of GNN models on a range of state-of-the-art protein function prediction tasks (see supplementary material of [92]).

Despite the good results obtained so far, it is still not clear if the graph representation of a structure can aid in predicting protein function. In particular, the study in [69] shows that introducing topological information and applying a GCN architecture on the protein graph may not improve sequence-to-function predictions. Moreover, one experiment showed that using different versions of the original protein graph, namely, a shuffled graph (residues at different locations), a disconnected graph (no edges), a fully connected graph (all residues connected) and a sequential-graph (covalent bonds only) produces similar results, indicating that the structural protein graph priors do not affect the final predictions. Therefore, more in-depth studies are needed to experimentally validate the use of graph learning in protein function prediction.

## Protein design

Protein design seeks to support the manual engineering of proteins for specific functions, without relying on evolutionary mechanisms [93]. More formally, given a protein structure, the goal is to find a plausible underlying amino-acid sequence (inverse protein-folding problem) [74]. Advances in protein structure prediction [8,9] have motivated corresponding advances in protein design. Taking advantage of protein structural data, GRL methods have been used to predict effective protein sequences. An example of such a method is ProteinSolver [71], which formulates protein design as a constraint satisfaction problem. To overcome computational complexity, it employs a GNN architecture to encode the constraint graph, where nodes are the amino-acids in the protein sequence, and the edges are the constraints that stem from the protein contact map. The authors show that ProteinSolver can be successful in generating novel protein sequences for a predetermined fold. MimNet improves on ProteinSolver on all CASP 7-12 challenges by using a specialized GCN-based architecture that solves the protein structure and protein design problems in tandem [73]. This is achieved by it’s bi-directional neural network architecture, which allows it to train for both protein folding and protein design simultaneously, doubling the amount of data available. Lastly, the *Structured Transformer* introduced in [74], inspired by machine translation tasks, ‘translates’ an input protein structure to a sequence profile. Among other results, it achieves better per-residue perplexities than purely sequence models, showcasing the power of GNN-based protein designed models that are conditioned on the structure.

GNNs have also shown potential in aiding protein design in other ways. In [94], a variation of GNNs [95] is employed to learn from molecular dynamics simulation data to infer protein allostery. The model proposed correctly infers the pathways that can mediate the allosteric communications between two binding sites. These results can aid protein design by mutating the amino acids in the allosteric pathways, in order to change protein function. This, combined with the ability to use inverse architectures to guide protein design [69,73], showcases the existing fertile ground for future protein design studies.

## Conclusion

This paper examines the potential of GRL to incorporate protein structures in various biological problems. The increasing availability of structural datasets and the recent explosion of work in the GRL field have increased the potential of exploiting structural priors in downstream proteomics tasks. Such structural information could be the primary reason why graph-based models edge out their sequence-based counterparts in regards to the studies discussed.

The GRL field was introduced essentially in the last 5 years, and, ever since then, it has made a huge impact on multiple scientific fields. Given the preliminary results from proteomics-related studies that employ GRL, its use to bioinformatics and computational biology is expected to inspire much structural proteomics research in upcoming years.

## Summary

- The increased availability of protein structural data has enabled the use of graph deep learning approaches in structural proteomics.
- GRL approaches have already been successfully employed in a multitude of tasks in many different domains/fields.
- In structural proteomics, GRL approaches have been used in protein–ligand interaction, protein function prediction and protein design, among others.
- Results from the studies discussed indicate that there is potential in using GRL methods together with ever-increasing protein structure data for a multitude of structural proteomics tasks.

## Abbreviations

CNNs: convolutional neural networks
DL: deep learning
GAT: graph attention network
GCN: graph convolutional network
GRL: graph representation learning
ML: machine learning
PDB: Protein Data Bank
